# Study on the Stability of Cu-Ni Cluster Components and the Effect of Strain on Its Structure

**DOI:** 10.3390/ma16216952

**Published:** 2023-10-30

**Authors:** Xiaochuan Zeng, Cuizhu He, Xuejun Li, Qiaodan Hu

**Affiliations:** 1 Institution of Advanced Materials and Solidification, Shanghai Jiao Tong University, Shanghai 200240, China; xie25@126.com; 2 State Key Laboratory of Nuclear Power Safety Monitoring Technology and Equipment, China Nuclear Power Engineering Co., Ltd., Shenzhen 518172, China; xinyu11.love@163.com (C.H.); lxj117@163.com (X.L.)

**Keywords:** solute cluster, molecular dynamics, shear strain, dislocation

## Abstract

Solute clusters are one of the important mechanisms of irradiation embrittlement of ferritic steels. It is of great significance to study the stability of solute clusters in ferritic steels and their effects on the mechanical properties of the materials. Molecular dynamics was used to study the binding energy, defect energy, and interaction energy of 2 nm-diameter Cu-Ni clusters in the ferritic lattice, which have six categories of Cu-Ni clusters, such as the pure Cu cluster, the core–shell structural cluster with one layer to four layers of Ni atoms and the pure Ni cluster. It was found that Cu-Ni clusters have lower energy advantages than pure Ni clusters. Through shear strain simulation of the three clusters, the structure of 2 nm diameter clusters does not undergo phase transformation. The number of slip systems and the length of dislocation lines in the cluster system are positively correlated with the magnitude of the critical stress of material plastic deformation.

## 1. Introduction

As a typical steel material for nuclear reactor pressure vessels (RPV), the hardening leading to embrittlement of low-alloy ferritic steel under irradiation has always been a key factor challenging the operation safety of the material [1]. Solute clusters are an important mechanism for the irradiation hardening-induced embrittlement of ferritic steels. The clusters of Cu-rich atoms are the first nanocrystalline precipitates found widely in ferritic steels [2,3,4], while the clusters of Ni-rich atoms are considered to be the main factor for the material embrittlement in the later period of irradiation, which is always called the late blooming phase [5,6]. Some scholars have found that there is irradiation synergistic effect between Cu and Ni. During the irradiation environment, Ni, Mn and other rare elements tend to segregate on the surface of Cu-rich precipitates, forming Cu-Ni/Mn core–shell structure [7,8,9,10], and the Cu-Ni/Mn core–shell complex clusters are the main precipitating phase composition of modern low-Cu-content RPV steels. The embrittlement mechanism of the precipitated phase is that it hinders the slip of dislocation in the lattice, resulting in an increase in yield strength and a decrease in toughness of the RPV steels [11,12]. The diameter of precipitated Cu-Ni clusters under irradiation is approximately equal to 2 nm, and the initial solute clusters have a body-centered cubic (bcc) structure with the matrix [13,14,15].

In the exploration of the formation and evolution process of solute complex precipitates, many experts have used computational methods to carry out research [16,17,18,19], but there have been few studies on the proportional distribution of elements in the initial stage of the stable nucleation of Cu-Ni clusters. In this paper, typical Cu-Ni complex clusters are taken as the research object. The stable energy of Cu-Ni core–shell clusters with different components in bcc-Fe was studied, and the binding energy, defect formation energy, and interaction energy of the system were analyzed.

The main function of cluster in the deterioration of RPV steel is to prevent dislocation slip and cause material property embrittlement. In the calculation of molecular dynamics, Terentyev and Granberg et al. [20,21,22,23] studied the shear interaction between various known dislocations and clusters in bcc-Fe. The RPV steels are often affected by external strains (such as vacancy clusters, interstitial atomic clusters, and grain boundary misalignment) during operation [24]. The current investigations mainly focused on the calculation of the effect of strain on primary knock-on atom (PKA) defects [24,25,26], but there are few studies on the change in clusters and the evolution of system defect characteristics under strain conditions, which is of great significance for understanding the irradiation embrittlement mechanism of RPV steels. In the second part of this paper, based on the study of Cu-Ni complex clusters, molecular dynamics is used to calculate the evolution of cluster structure and the interaction mechanism between dislocation and cluster under the shear strain.

## 2. Computational Details

### 2.1. System Stability Calculation

In this investigation, the classical molecular dynamic simulations are performed utilizing the LAMMPS software package, 23Jun22 version [27]. In order to describe the interaction between Fe-Cu-Ni ternary alloys in this study, the Zhou–Johnson–Wadley force field in the embedded-atom method (EAM) potential was adopted [28]. A three-dimensional periodic boundary was applied in the model construction, and the basic unit of the system was bcc α-iron lattice. The entire supercell system contains 25 × 25 × 25 lattice units with a total of 31,250 atoms. A small system can be used to achieve a long-time simulation.

**Figure 1:**
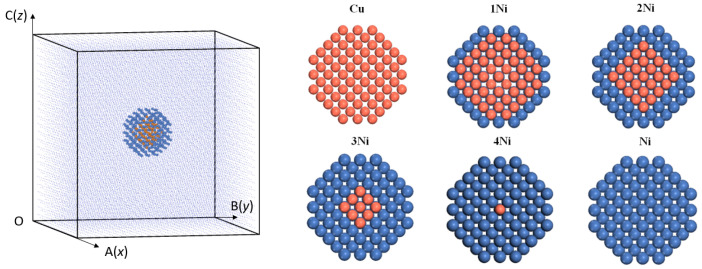
Cluster structures with different Cu-Ni proportions (yellow spheres are Cu atoms, blue spheres are Ni atoms), 1Ni is the core–shell structure of one layer of Ni atoms, 2Ni is the core–shell structure of two layers of Ni atoms, 3Ni is the core–shell structure of three layers of Ni atoms, and 4Ni is the core–shell structure of four layers of Ni atoms.

There is a Cu-Ni core–shell composite cluster with a diameter of about 2 nm in the center of the system, and the cluster is coherent with the matrix. In order to study the stable composition of clusters with different Cu and Ni ratios, the radial distribution of chemical composition of composite clusters in reference [10] was investigated. In the center of the simulation box, a sphere with a 2 nm diameter was selected, and all Fe atoms in the sphere were replaced by Ni atoms. In this way, the Ni cluster model was constructed with five layers of Ni atoms in the system. The four layers of atoms in the center of Ni cluster were replaced by Cu atoms, and the 1Ni cluster model was constructed. The three layers of atoms in the center of Ni cluster were replaced by Cu atoms, and the 2Ni cluster model was constructed. Rely on the same approach, the 3Ni cluster model and the 4Ni cluster model are constructed, respectively. The Cu cluster model is constructed by replacing all Ni atoms in the Ni cluster model. Therefore, all Cu clusters, 1Ni clusters with one layer of Ni shell covering the Cu core, 2Ni clusters with two layers of Ni shell covering the Cu core, 3Ni clusters with three layers of Ni shell covering the Cu core, 4Ni clusters with four layers of Ni shell covering the Cu core, and all Ni clusters are constructed. The cluster architecture is shown in Figure 1, and the number of atoms is shown in Table 1.

After the cluster models with different Cu-Ni ratios were constructed, the conjugate gradient method was used to optimize the geometric structure. The energy convergence criterion of the system was ≤10^−4^ kcal/mol, and the convergence criterion of the interaction force was ≤0.005 kcal/mol/A. Then, dynamic relaxation was performed at 300 K under the NPT ensemble with a time step of 1.0 fs. The system reached a relatively stable state after a cumulative time of 500 ps, and finally, the energy of the system was calculated. After dynamic relaxation, the binding energy, defect formation energy and interaction energy of the system are calculated by using the energy of the stable state, so as to find the component proportion of the cluster elements of the stable system model.

### 2.2. Calculation of Shear Strain Dynamics of Clusters

In order to compare the structure and energy changes in Cu-Ni clusters with different components in the shear deformation process, the system of pure Cu cluster, 2Ni cluster and pure Ni cluster in Section 2.1 was selected to apply continuous strain for the shear strain simulation. The dynamics of the system was simulated at 300 K under the NPT ensemble. The simulation temperature of 300 K was selected on the basis of the temperature of the RPV hydraulic test, during which the RPV steels experience the maximum stress throughout the operation life. Due to the three-dimensional symmetry of the system, the direction of the applied shear stress would not affect the dynamic results. In this paper, the shear stress was in the direction of OB (*y*-axis), and the shear process led to the change in γ Angle. The selection of shear rate refers to the influence factors of shear rate [29]. In this kinetic simulation, the shear rate is 5 × 10^8^ S^−1^, the time step is 1.0 fs, the cumulative time is 500 ps, and one frame of data is output every 1 ps to simulate the interaction process between cluster and strain. The formation and evolution of defects and structural changes in the process of shear dynamics simulation were analyzed using the OVITO software package, 3.0.0 version [30].

## 3. Results and Discussion

### 3.1. Energy Comparison of Different Systems

When calculating the binding energy, defect formation energy and interaction energy of the system, the energy acquisition of single Fe atom is obtained through the energy calculation of bcc iron lattice, and the energy acquisition of single Cu and Ni atoms is obtained by replacing the atoms in bcc-Fe lattice with Cu atoms and Ni atoms, respectively, to form a bcc-Cu lattice and bcc-Ni lattice. Then, the energy calculation of the three kinds of atoms in the bcc lattice is obtained using the energy calculation as follows (Table 2).

Firstly, the binding energy of clusters is calculated, which indicates the size of the attractive force between atoms in the system. The smaller binding energy of clusters with different Cu-Ni ratios means that the clusters are easier to form. The calculation formula of binding energy is as follows [31]:

(1) $$E_{binding\;energy}=\left[E\left({Fe}_{31,250-x-y}{Cu}_{x}{Ni}_{y}\right)-\left(31,250-x-y\right)E\left(Fe\right)-xE\left(Cu\right)-yE\left(Ni\right)\right]/31,250$$

where *E*(*Fe*_31,250−*x*−*y*_*Cu_x_Ni_y_*) is the total energy of system *Fe*_31,250−*x*−*y*_*Cu_x_Ni_y_*, and *E*(*Ni*), *E*(*Cu*), and *E*(*Fe*) are the energy of a single atom in an elemental system of bcc-Fe, bcc-Cu, and bcc-Ni, respectively.

Defect formation energy represents the difficulty of system defect formation. The smaller the defect formation energy is, the easier the type of defect is formed and the more stable the system is. The calculation formula of defect formation energy is given in reference [32]:

(2) $$E_{defect}=E\left({Fe}_{31,250-x-y}{Ni}_{x}{Cu}_{y}\right)+\left(x+y\right)E\left(Fe\right)-E\left({Fe}_{31,250}\right)-xE\left(Ni\right)-yE(Cu)$$

where *E*(*Fe*_31,250−*x*−*y*_*Ni_x_Cu_y_*) is the total energy of system *Fe*_31,250−*x*−*y*_*Ni_x_Cu_y_*, *E*(*Fe*_31,250_) is the total energy of system *Fe*_31,250_, and *E*(*Ni*), *E*(*Cu*), and *E*(*Fe*) are the energy of a single atom in an elemental system of bcc-Fe, bcc-Cu, and bcc-Ni, respectively.

**Figure 2:**
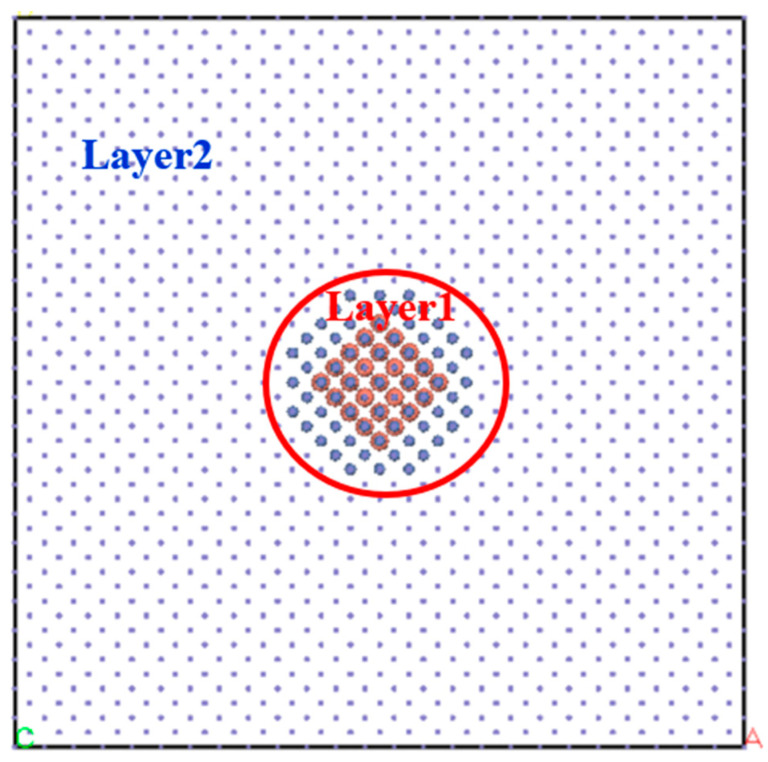
Structural model of the *Fe*_31,250−*x*−*y*_*Ni_x_Cu_y_* system, the yellow spheres in Layer 1 are Cu atoms, the blue spheres in Layer 1 are Ni atoms and the blue dots in Layer 2 are Fe atoms.

The interaction energy represents the interaction force between two classes of substances, and the larger its absolute value is, the stronger the interaction between them. In order to calculate the interaction energy between Cu-Ni clusters and Fe matrix in the system, the energy of Fe matrix and the individual energy of clusters are subtracted from the total energy of the system. The calculation formula is as follows:

(3) $$E_{interaction}=E\left({Fe}_{31,250-x-y}{Ni}_{x}{Cu}_{y}\right)-E\left(Layer1\right)-E(Layer2)$$

where *E*(*Fe*_31,250−*x*−*y*_*Ni_x_Cu_y_*) is the total energy of system *Fe*_31,250−*x*−*y*_*Ni_x_Cu_y_*, *E*(*Layer*1) is energy of Layer1 in Figure 2, and *E*(*Layer*2) is the energy of Layer2 in Figure 2.

**Figure 3:**
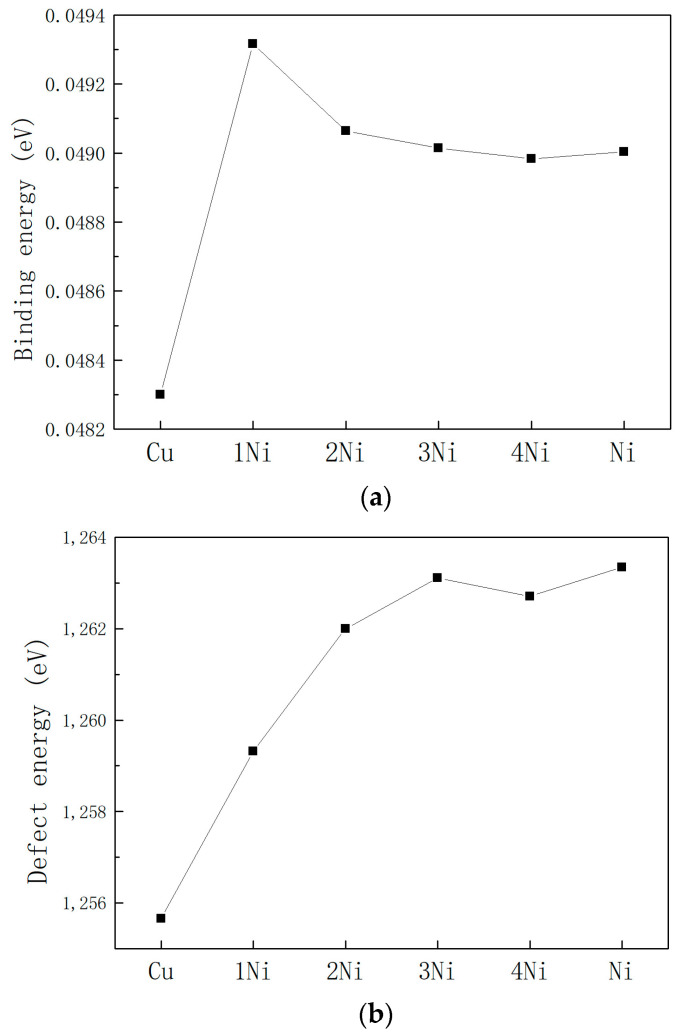

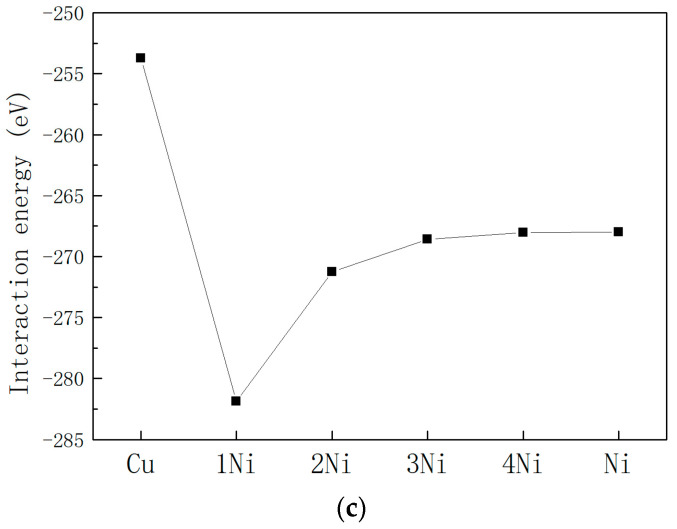
Comparison of binding energy (**a**), defect formation energy (**b**), and interaction energy (**c**) of the cluster system under different Cu-Ni ratios (Cu represents the full Cu cluster, 1–4Ni represents the Cu-Ni cluster with 1–4 layers of Ni, and Ni represents the full Ni cluster).

The binding energy, defect formation energy and interaction energy of the six cluster systems in Figure 1 were calculated by the above three equations based on the molecular dynamics simulation results, and the calculation results are shown in Figure 3.

Figure 3a shows the binding energy curves of systems with different Cu-Ni ratios. It can be seen from the figure that the binding energy of all systems is positive, which indicates that the formation of Cu-Ni clusters is an endothermic process from the perspective of thermodynamics. Comparing the binding energy values of different Cu-Ni ratio systems, it can be seen that the binding energy value of the pure Cu cluster system is the smallest, indicating that the pure Cu cluster formation process requires less heat absorption and is easier to form. The addition of Ni element increases the binding energy of the system, and the binding energy of the system tends to be stable with the increase in the percentage of Ni atoms.

Figure 3b shows the defect formation energy curves of systems with different Cu-Ni ratios. It can be seen from the figure that the defect formation energy of all systems is positive, which is also an endothermic process from the perspective of thermodynamics. Comparing the defect formation energy values of clusters with different Cu-Ni ratios, the pure Cu cluster system has the smallest defect formation energy and is the easiest to form, which is consistent with the analytical conclusion of binding energy. With the increase in Ni content, the defect formation energy gradually increases, and the pure Ni cluster has the largest defect formation energy, which also reflects that compared with the pure Ni cluster, the addition of the Cu core can reduce the defect formation energy of the cluster.

Figure 3c shows the interaction energy of the system with different Cu-Ni proportions. Based on the calculation Formula (3), it can be approximately regarded as the strength of the interaction between the Fe matrix and the Cu-Ni composite cluster in the system. From the data in the figure, the absolute value of the interaction energy of the pure Cu cluster system is the smallest, indicating that the interaction between the Fe matrix and the Cu core is the weakest. When the number of Ni atomic layers is 1, the absolute value of the interaction energy is the largest. As the number of Ni atomic layers increases, the absolute value of the interaction energy gradually decreases.

According to the calculation results of binding energy, defect formation energy, and interaction energy, it is finally determined that when the central cluster is all Cu atoms, the system has the lowest energy and is easiest to form, which is consistent with the experimental observation that the Cu precipitate phase is formed first [3,4,33]. The energy of 1Ni, 2Ni, 3Ni, 4Ni, and pure Ni clusters (including binding energy, defect formation energy, and interaction energy) changes monotonically. When the number of Ni atomic layers reaches two, the binding energy and interaction energy change very little, but compared with pure Ni clusters, the composite clusters with Cu as the core reduce the defect formation energy of the system. This indicates that the formation of Cu-Ni composite clusters is feasible in terms of energy.

### 3.2. Strain Energy Calculation for Different Clusters Systems

**Figure 4:**
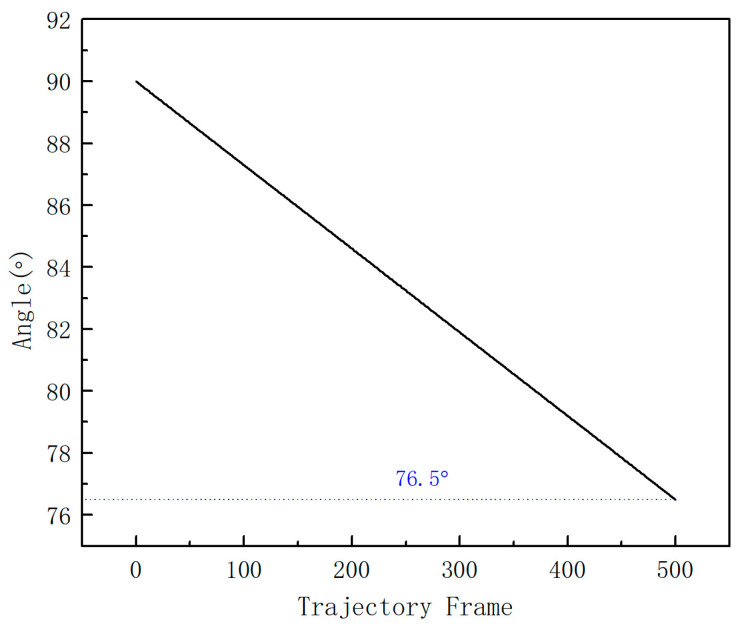
Curve of the γ Angle in the system as a function of trajectory frame, the dotted line indicates that the final γ Angle is 76.5°.

**Figure 5:**
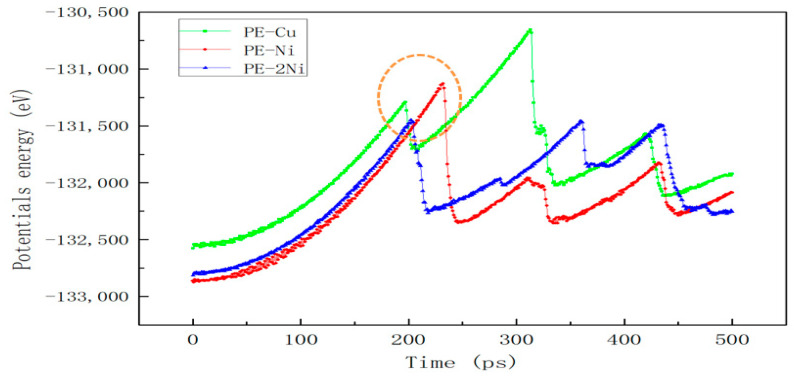
Plots of potential energy changes for different systems under shear strain; green line is Cu cluster, red line is Ni cluster, and blue line is 2Ni cluster. The first peak of the three curves is highlighted in yellow circle.

In order to study the change process of Cu-Ni cluster defects under the shear strain, a pure Cu cluster system, a 2Ni composite cluster system, and a pure Ni cluster system were selected for the shear strain simulation, and the maximum change in strain γ Angle of the system after 500 ps was 13.5°. In Figure 4, the γ Angle changes from 90° to 76.5°, that is, a difference of 13.5°. Figure 5 shows the curve of the system potential energy as a function of time during the shear strain. With the increase in strain rate, the energy of the system gradually rises at the beginning, and then drops sharply after reaching the apex, and then the energy fluctuates like waves. By comparing the energy peak (EP) of the three systems, EP(Cu) > EP(Ni) > EP(2Ni), according to E = F × S, since the strain rate of the system is constant, the stress of the system is positively correlated with the potential energy at this time; that is, the pure Cu cluster has the largest strain resistance, the pure Ni cluster has the second largest, and the 2Ni composite cluster has the smallest. That is, the pure Cu cluster produces the largest resistance to the shear strain, and the 2Ni composite cluster has the smallest stress to the strain. The magnitude of shear stress is positively correlated with cluster hardness [23].

**Figure 6:**
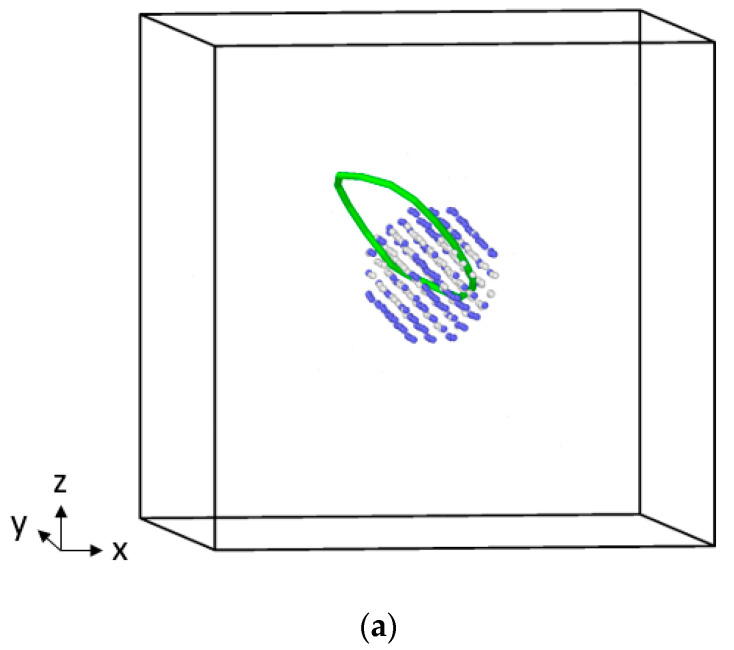

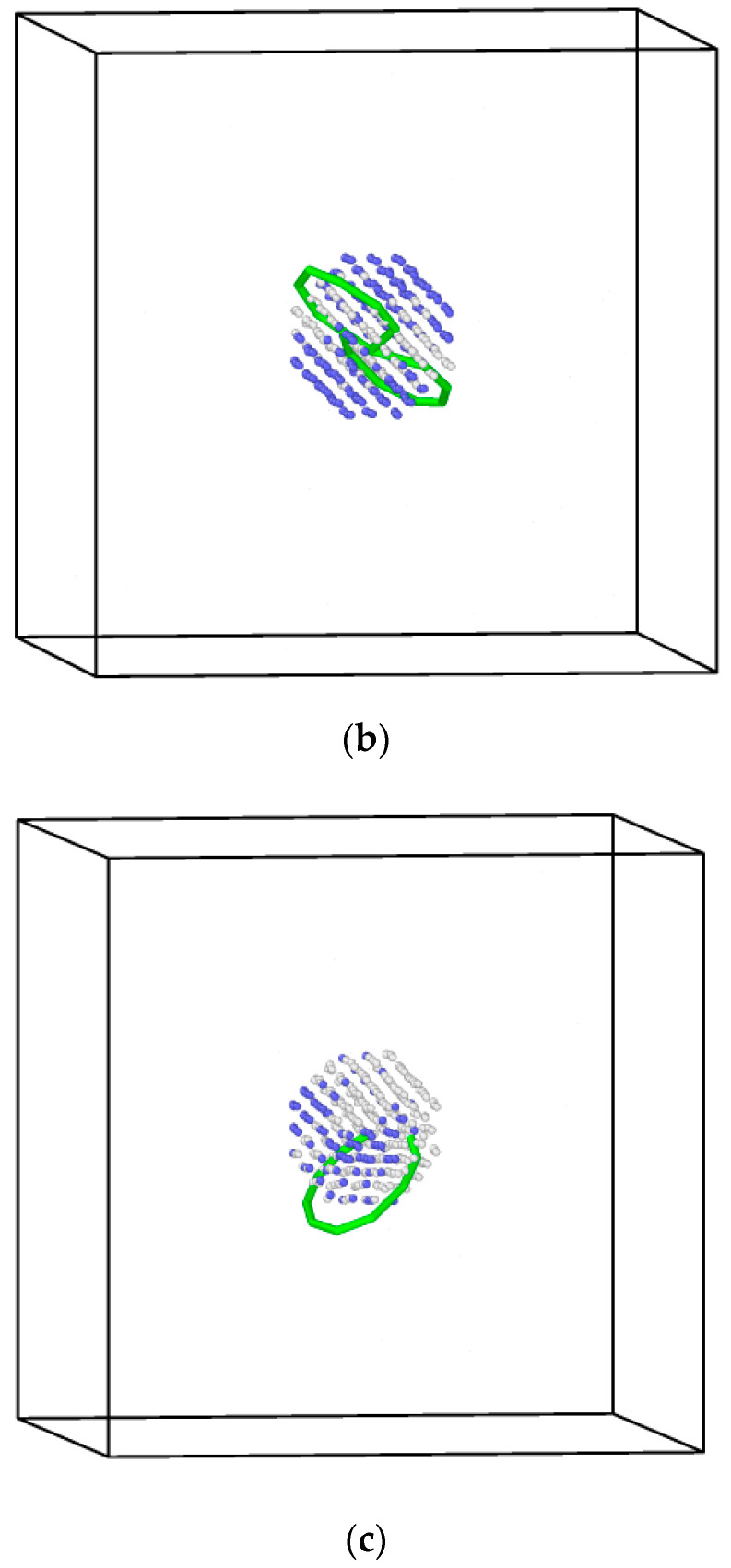
Structure of clusters in different systems at the first energy peak ((**a**) represents pure Cu cluster, (**b**) represents 2Ni cluster, (**c**) represents pure Ni cluster), green is dislocation line, blue atom is the bcc structure, and white atom is disordered structure.

According to the energy change of the system, elastic deformation is dominant in the initial stage of the shear as that the system structure has not changed significantly all the time, and only the γ Angle of the *z*-axis changed. When the system energy reaches the first peak point, it means that the critical stress point is reached. Then the energy drops sharply and the system undergoes plastic deformation, which indicates that the microstructure changes greatly. In the structural analysis of the first peak point of the curve, it was found that after shear deformation of the result, part of the cluster structure maintained the bcc structure, and part of the cluster lattice showed disordered arrangement, and no new structure (phase) was generated. The results are shown in Figure 6. However, it can be seen in Figure 6 that an obvious dislocation ring is generated around the cluster, and the Burgers vector of the dislocation is 1/2<111>, which is consistent with the slip direction of the dislocation in the bcc structure [34]. For the 2Ni cluster, an 8-shaped double dislocation ring structure appears throughout the cluster center, which shows that the number of dislocation rings is related to the misfit type of the system. However, the stress differences of the three clusters at the first peak point are obviously positively correlated with the misfit values (0.34 and 0.45) between different clusters and iron matrix and the length of dislocation lines [35]. The longer of the dislocation line, the more atoms can slip, the lower the stress value of the system. The simultaneous dislocation loop surrounding the cluster presents a twisted lasso before and after plastic deformation, and the resulting sliding of non-crystallographic planes is an atypical Orowan bypassing mechanism [36].

**Figure 7:**
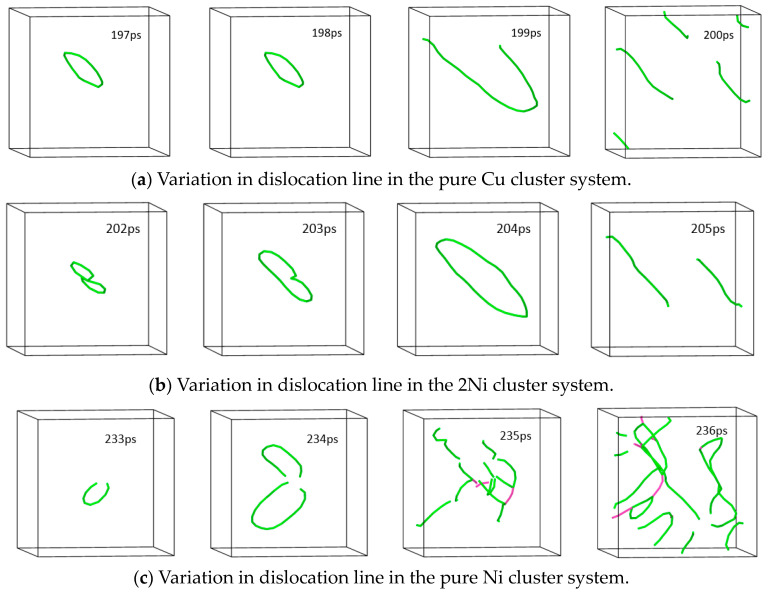
Changes in dislocation lines in three systems within three trajectory frames after the stress critical point (Burgers vector b = 1/2<1 1 1> for green dislocation line; Burgers vector b = <0 0 1> for pink dislocation line).

When the shear strain passes the critical stress point, the main structure of the cluster system still maintains the bcc lattice structure, and no new phase is generated. Figure 7 shows the changes in dislocation lines in the three systems of pure Cu cluster, 2Ni composite cluster, and pure Ni cluster at the critical stress point (corresponding time points are 197 ps, 202 ps, and 233 ps, respectively) and the next three frames. When the energy of the system reaches the critical stress point, with the increase in the stress variable, the dislocation ring in the Cu cluster system and Ni cluster system diffuses outward around the cluster, and the size becomes larger until it breaks and generates multiple dislocation lines in the system, and the cluster becomes the source of dislocation proliferation in the system. The number of dislocation lines increases the number of slip systems and makes the stress level of the system decrease rapidly. With the annihilation of the dislocation lines at the grain boundaries and the lack of new slip systems in the system, the stress level will gradually increase until a new dislocation line appears, at which point the stress level reaches the next peak. This happens repeatedly, causing the stress level to fluctuate.

Finally, after the maximum 13.5° shear deformation of the system, the structures of the clusters show that most of the atoms in the three systems still maintain the bcc structure. According to reference [37], when the cluster size is less than 3 nm in ferrite steel, the phase transition will not occur due to the small defect size and lack of driving force. In the subsequent deformation, the dislocations in the system are in a repeating state of proliferation–annihilation, and the significance of dislocation line research is no longer significant.

## 4. Conclusions

Through the data analysis of binding energy, defect formation energy, and interaction energy of the cluster system with different Cu-Ni ratio, it is confirmed that when the cluster comprises the full Cu atoms, the system has the lowest capacity and is the easiest to form. This phenomenon is consistent with the experimental observation that the Cu precipitate phase is formed first. The formation of Cu-Ni composite clusters has a lower energy advantage than the pure Ni clusters.

In the shear dynamics simulation and structural analysis of the Cu-Ni clusters of the three components, it is found that the structure of the 2 nm size bcc structure cluster does not change significantly in the shear strain, but the cluster can be used as the dislocation source of the ferrite steel-plastic strain, and the length of the dislocation line will affect the critical stress of the system. The finding that the critical stress for plastic deformation of the Cu-Ni composite cluster system is lower than that of pure Cu and pure Ni clusters can be used as an explanation for the difference in hardening degree caused by different cluster types in RPV steel.

## Figures and Tables

**Table 1:** The number of Fe-Cu-Ni atoms in different systems.

Category	Number of Fe Atoms	Number of Cu Atoms	Number of Ni Atoms
Cu	30,911	339	0
1Ni	30,911	169	170
2Ni	30,911	65	274
3Ni	30,911	15	324
4Ni	30,911	1	338
Ni	30,911	0	339

**Table 2:** Energies of Fe, Cu, and Ni atoms in the bcc lattice.

Type of Atom	Fe	Cu	Ni
Energy/eV	4.254	3.449	4.318

## Data Availability

Not applicable.
